# Phospholipid transfer protein activity in two cholestatic patients

**DOI:** 10.1590/S1516-31802004000400009

**Published:** 2004-07-01

**Authors:** Eliana Cotta de Faria, Adriana Celeste Gebrin, Wilson Nadruz, Lucia Nassi Castilho

**Keywords:** Cholestasis, Lipoprotein X, Phospholipids, Apolipoprotein A-I, Hypercholesterolemia, Lipoproteína X, Fosfolipídeos, Apolipoproteína A-I, Hipercolesterolemia

## Abstract

**CONTEXT:**

Plasma phospholipid transfer protein mediates the transfer of phospholipids from triglyceride-rich lipoproteins, very low density lipoproteins and low density lipoproteins to high density lipoproteins, a process that is also efficient between high density lipoprotein particles. It promotes a net movement of phospholipids, thereby generating small lipid-poor apolipoprotein AI that contains particles and subfractions that are good acceptors for cell cholesterol efflux.

**CASE REPORT:**

We measured the activity of plasma phospholipid transfer protein in two cholestatic patients, assuming that changes in activity would occur in serum that was positive for lipoprotein X. Both patients presented severe hypercholesterolemia, high levels of low density lipoprotein cholesterol and, in one case, low levels of high density lipoprotein cholesterol and high levels of phospholipid serum. The phospholipid transfer activity was close to the lower limit of the reference interval.

To our knowledge, this is the first time such results have been presented. We propose that phospholipid transfer protein activity becomes reduced under cholestasis conditions because of changes in the chemical composition of high density lipoproteins, such as an increase in phospholipids content. Also, lipoprotein X, which is rich in phospholipids, could compete with high density lipoproteins as a substrate for phospholipid transfer protein.

## INTRODUCTION

Plasma phospholipid transfer protein mediates the transfer of phospholipids from triglyceride-rich lipoproteins, very low density lipoproteins and low density lipoproteins to high density lipoproteins, a process that is also efficient between high density lipoprotein particles. It promotes a net movement of phospholipids, thereby generating small lipid-poor apolipoprotein AI that contains particles and subfractions that are good acceptors for cell cholesterol efflux. We measured the activity of plasma phospholipid transfer protein in two cholestatic patients, assuming that changes in activity would occur in serum that was positive for lipoprotein X.

## CASE REPORT

**Figure 1:**
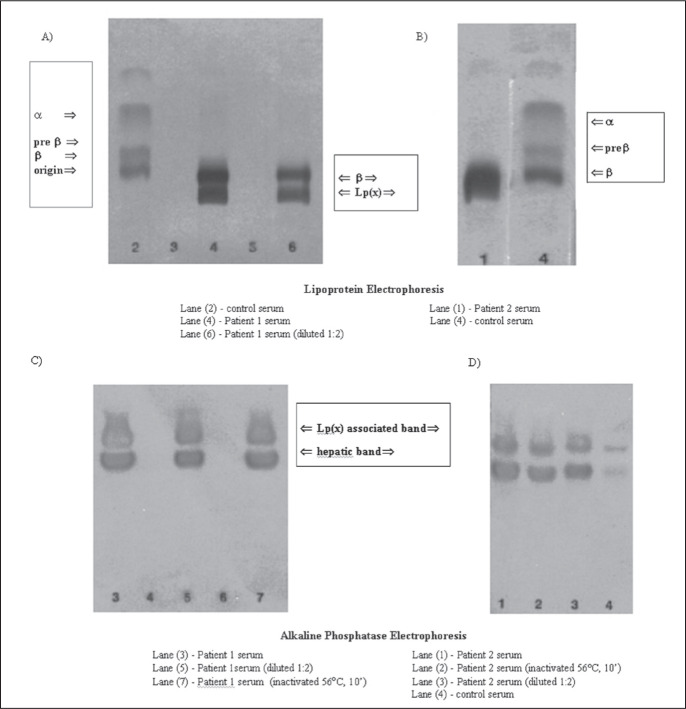
Serum lipoprotein and alkaline phosphatase electrophoresis (agarose gel).

Patient 1 was a 58-year-old man who presented primary sclerosing cholangitis shown by retrograde endoscopic cholangiopancreatography and ulcerative colitis shown by colonoscopy. A liver biopsy revealed moderately active disease of the biliary tract. He was released from hospital under cholestyramine treatment. The results of serum electrophoresis are shown in Figure 1 (panels A and C).

Patient 2 was a 40-year-old woman. Liver biopsy revealed the presence of anti-mitochondrion antibodies consistent with a diagnosis of primary biliary cirrhosis. She was submitted to cholestyramine and fat-soluble vitamin supplementation. No established atherosclerotic disease was detected in either patient, as often found.^1^

Both patients presented severe increases in the levels of bilirubin, hepatic and biliary enzymes (not shown). In Table 1, the changes in the lipid parameters and the phospholipid transfer protein activity are shown.

**Table 1:** Serum lipids, lipoproteins, apolipoproteins (mg/dl) and phospholipid transfer protein activity (% phospholipid transfer/hour) in two cholestatic patients

Patients	Cholesterol*	Triglycerides*	Low density lipoprotein cholesterol*	High density lipoprotein cholesterol*	Phospholipids†	Phospholipid transfer protein activity ‡	Apolipoprotein AI §	Apolipoprotein B ||
1	995	187	943	22	1156	undetectable	57	170
2	731	244	675	49	640	2	< 25	231

(*) *reference range in accordance with the National Cholesterol Education program Adult Treatment Panel (NCEP ATP III) recommendations;*

(†) *reference range = 150 to 250 mg/dl;*

(‡) *reference range = 11 ± 2 % of phospholipid transfer/hour, with reference interval values taken from a healthy control population, n = 47;*

(§) *reference range = 94 to 199 mg/dl;*

(||) *reference range = 40 to 109 mg/dl.*

In Figure 1, the lipoprotein electrophoresis (Beckman Paragon) confirmed the presence of lipoprotein X in both patients (panels A and B), revealed by a fraction that is more cathodic than low density lipoprotein, located near the origin. Alkaline phosphatase isoenzyme electrophoresis (panels C and D) showed a band that was suggestive of an association between biliary alkaline phosphatase and lipoprotein X.

## DISCUSSION

Xanthomas and markedly raised plasma cholesterol levels coexist in chronic liver disease in the presence of lipoprotein X.^1^ Lipoprotein X is not taken up by the liver lipoprotein receptors (B, E and low density lipoprotein receptor related protein) and therefore can not exert a feedback control on hepatic cholesterol biosynthesis. It is removed from the circulation by the reticuloendothelial system.

The reduction in the activity of phospholipid transfer protein in these patients was probably due to changes in the chemical composition of the lipoproteins, such as phospholipid enrichment, especially in relation to high density lipoprotein.^2,3^ In addition to this, lipoprotein X, which is also rich in phospholipids, could compete with high density lipoprotein as a substrate for phospholipid transfer protein. The reduced phospholipid transfer protein activity shown here could also explain the low levels of high density lipoprotein cholesterol found in one of these patients.

We suggest that studies of lipoprotein metabolism in such patients are extremely important because they might further increase our knowledge of the underlying mechanisms involved in dyslipidemia and the absence of atherosclerosis in the presence of lipoprotein X.^4^
